# Nurses' knowledge regarding the early identification of acute kidney injury

**DOI:** 10.1186/cc10956

**Published:** 2012-03-20

**Authors:** S Agege Lobo, RM Matheus

**Affiliations:** 1 Faculdade de Medicina de São José do Rio Preto - FAMERP, São José do Rio Preto, Brazil

## Introduction

The objective was to evaluate nurses' knowledge on the early identification of acute kidney injury (AKI) in an ICU, inpatient care unit, and emergency unit.

## Methods

This was a multicenter, prospective, longitudinal study. The study population included 216 nurses who work in the ICU, inpatient care unit, and emergency unit at six public and private hospitals. Data collection was performed from October 2010 to February 2011 using a 10-question questionnaire related to prevention, diagnosis, and treatment of AKI.

## Results

Data showed that 81.7% of the nurses gave correct answers regarding the association of urine volume rate in the identification of AKI; 57.2% did not know how to identify the clinical manifestations of AKI; 67.1% made a mistake by answering that the subtle increase of creatinine has no great impact on a mortality rate; 66.8% answered the question incorrectly on measures to prevent AKI; 60.4% were correct when they answered that the use of loop diuretics in the prevention of AKI is not recommended; and 92.5% said they had no knowledge of the Acute Kidney Injury Network classification.

## Conclusion

The results showed that most nurses do not have enough knowledge for the early identification of AKI. This highlights the importance of training programs for nurses who work at hospital units, with the purpose of developing professional competences and aptitudes regarding both prevention and detection of AKI.

